# Internet-based interventions to support recovery and self-management: A scoping review of their use by mental health service users and providers together

**DOI:** 10.1186/s12888-019-2153-0

**Published:** 2019-06-20

**Authors:** Anne Williams, John Farhall, Ellie Fossey, Neil Thomas

**Affiliations:** 1 Department of Occupational Therapy, Social Work and Social Policy, La Trobe University, Melbourne, Victoria Australia; 2 Department of Health Professions, Swinburne University of Technology, Hawthorn, Victoria Australia; 3 Department of Psychology and Counselling, La Trobe University, Melbourne, Victoria Australia; 4 NorthWestern Mental Health, Melbourne Health, Melbourne, Melbourne, Victoria Australia; 5 Department of Occupational Therapy, Monash University, Frankston, Victoria Australia; 6 Living with Disability Research Centre, La Trobe University, Melbourne, Victoria Australia; 7 Centre for Mental Health, Swinburne University of Technology, Hawthorn, Victoria Australia; 8 Monash Alfred Psychiatry Research Centre, Melbourne, Victoria Australia

**Keywords:** Internet, mental health, mental health services, psychosis, recovery-oriented practice, self-management, severe mental illness

## Abstract

**Background:**

Internet-based interventions can make self-management and recovery-oriented information and tools more accessible for people experiencing severe mental illness, including psychosis. The aim of this scoping review was to identify and describe emerging joint uses of these Internet-based interventions by service users experiencing psychosis and mental health workers. It also investigated how using these Internet-based interventions influenced interactions between service users and workers and whether recovery-oriented working practices were elicited.

**Methods:**

A scoping review method was used. Iterative review stages included identifying the review question, a comprehensive search including searching six electronic databases to locate relevant studies, selecting studies, charting the data, and collating and reporting the results. Rigour of the scoping review was enhanced by using an appraisal tool to evaluate the quality of included studies, and by using a published template for systematic description of interventions.

**Results:**

Fifteen papers about eleven Internet-based interventions that focused on self-management and/or recovery were identified. Interventions were web-based, mobile-device based, or both. The eleven interventions were used by service users either with their usual mental health workers, or with mental health workers employed in a research project. Emerging evidence suggested that jointly using an Internet-based intervention could support a positive sense of working together. However, mismatched expectations and poor integration of Internet-based interventions into service systems could also negatively influence interactions, leading to mistrust. The interventions demonstrated potential to elicit recognised recovery-oriented practices, specifically understanding service users’ values and supporting their goal striving.

**Conclusions:**

The use of Internet-based interventions focused on self-management and recovery in mental health services by service users and workers jointly demonstrates potential to support working together and recovery-oriented practice. Given that the quality of relationships is critical in recovery-oriented practice, greater focus on human support in Internet-based interventions is needed in future research and practice.

**Electronic supplementary material:**

The online version of this article (10.1186/s12888-019-2153-0) contains supplementary material, which is available to authorized users.

## Background

People who live with a diagnosis of a severe mental illness (SMI) often experience enduring mental, physical, social and economic impacts from their illness experience and are high users of mental health services [1]. This includes people described in the literature as experiencing schizophrenia and related diagnoses, affective mental illnesses with psychotic symptoms, and other psychotic disorders [1]. In line with a gradual reorientation of health services towards health promotion [2], mental health services targeted at people experiencing SMI increasingly aim to deliver services that move beyond clinical treatment and crisis management [3]. Current imperatives include supporting service users experiencing SMI in their individual recovery journeys [3–5] and enabling them to self-manage their physical and mental health [2]. Supporting service users in their personal recovery, defined as leading an individually determined meaningful and productive life in the context of living with a mental illness [6, 7], has become a mental health service priority [3]. The related focus on self-management repositions service users experiencing SMI as active participants in their treatment and provides tools that can empower them to have the confidence to manage their health over time, thus supporting their ongoing recovery journey [2].

Internet-based interventions have potential to enhance mental health services, including by supporting self-management and recovery among people experiencing severe mental illness [8]. This population is using the Internet and interested in Internet-based interventions [9–12]. Types of interventions delivered via the web and mobile devices include psychoeducation, illness monitoring and management tools, communication and decision-making tools and peer support [13–15]. For example, in their review of remotely delivered mHealth and eHealth interventions, Naslund et al. [15] included messaging services targeting illness self-management; websites with education, coping skills tools and peer support; and activity trackers connected to a smartphone app designed to increase physical activity. These interventions may be designed specifically for people experiencing SMI by incorporating simple visual designs and customised reading and abstract reasoning demands [15]. One way to categorize the broad range of Internet-based interventions currently available is as either web-based (for example, websites, discussion rooms and Internet diaries accessed via the Internet on a computer or other device), or mobile device-based (for example, text messaging or applications used on a smartphone or other mobile device) [13].

Numerous reviews indicate that Internet-based interventions are feasible and acceptable for people experiencing SMI [16–20], with promising signs of their efficacy [13, 17, 19]. Identified benefits of web-based and mobile-device based interventions include supporting users’ treatment engagement, building their self-management and coping skills, increasing their knowledge about their illness, improving symptom management and preventing relapse [8, 13–15]. These findings have been welcomed given the accessability, potential for anonymity and cheaper cost of delivering Internet interventions compared to interventions delivered face-to-face [13]. Internet interventions are expected to continue to develop and become more widely available to people experiencing SMI in the future [14, 15, 19, 21].

There are potential challenges to integrating Internet-based interventions for self-management and recovery into usual mental health services for people experiencing SMI. van der Krieke et al. [19] argue that a focus on symptom monitoring in many Internet-based self-management interventions makes a personal recovery approach difficult, by reproducing “an outdated paternalistic paradigm of patient-clinician interaction in which compliance and monitoring are the aim” (p. 46-47). Additionally, Villani and Kovess-Masfety [12] identified that people experiencing psychotic disorders may perceive that raising treatment information from the Internet with their doctor could negatively impact their relationship, if the doctor feels criticized. In considering future challenges, Álvarez-Jiménez et al. [14] propose that “Internet-based interventions for psychosis that are specifically designed to supplement existing mental health services and augment traditional relationships through online interaction… are likely to be the most beneficial” (p.742). However, others have noted that engaging service users [19, 21] and workers [21] in these interventions may be difficult. In sum, integrating Internet-based interventions into mental health services is likely to be complex, and attention will need to be paid to how relationships between service users and their mental health workers might best work in a digital environment.

The quality of relationships between service users and workers is core to recovery-oriented practice [22, 23] and to facilitating partnerships that support self-management [2], yet the place of professional supervision and support in Internet-based interventions is considered infrequently [13] and the impact of the interventions on working relationships has received little attention. One review by Strand et al. [24] considered the extent to which Internet-based interventions integrated into ongoing mental health care supported recovery-oriented practice domains as defined by Le Boutillier et al. [3], including recovery-promoting relationships. Their review included six interventions published up to May 2015, which were used with people experiencing long-term mental health problems. Strand et al. [24] concluded that while these interventions supported work in which recovery was personally defined by service users, it was less clear whether using the interventions supported recovery-promoting relationships or inspired hope [24]. Further investigation of how jointly using an Internet-based intervention can influence working relationships is therefore needed. The aim of this scoping review was to conduct a systematic search of the literature to identify and describe joint uses of Internet-based interventions focused on recovery and/or self-management, by service users experiencing SMI and mental health providers, and to explore how these interventions influence their interactions.

## Methods

### Scoping review methodology

A scoping review method involving five stages [25–27] was selected to explore the range of research activity in this area of mental health practice and to address questions that go beyond intervention effectiveness [26]. We also adopted Daudt et al.’s [28] recommendation to assess the quality of studies included in scoping reviews.

#### Stage 1: Identifying the review question

The review questions comprised:

1. What Internet-based interventions (web-based or mobile-device based) with a recovery and/or self-management focus are service users experiencing SMI, specifically psychosis, and mental health workers using within mental health services?
2. How does using these Internet-based interventions influence interactions between these service users and workers?
3. Does using Internet-based interventions focused on recovery or self-management elicit recovery-oriented working practices?

#### Stage 2: Identifying relevant studies

The literature search was guided by Atkinson et al. [29] to enhance its transparency. Six electronic databases were searched from 2005 to March 2016: The Cochrane Library; Embase (Ovid); Cumulative Index to Nursing and Allied Health Literature (CINAHL) (Ebsco); Computer Database (Gale); Medline (Ovid); and PsychINFO (Ovid). The search period reflected that earlier Internet-based interventions were designed for people experiencing anxiety and depression, not SMI [17, 19, 20]. A university librarian reviewed and approved the search strategy, followed by the first author conducting the database searches using the key words shown in Table 1, modified as necessary for specific databases.

**Table 1:** Search terms, inclusion and exclusion criteria

	Key search terms	Inclusion criteria	Exclusion criteria
Population	Severe mental illness Serious mental illness Schizophrenia Psychotic disorders Psychotic illness	Adults experiencing SMI including psychotic illness or mental illnesses with psychotic features	Solely high prevalence mental health conditions including anxiety, depression Solely bipolar disorder or major depression where presence of psychosis is unclear
Intervention	Internet-based intervention Internet web-based eHealth, mHealth Online	*Primary:* Service user and mental health worker engaged in the intervention, whether synchronously or asynchronously *Secondary:* Service user and research personnel in mental health service engaged in the intervention	Intervention does not engage both service user and mental health worker or research personnel Stand-alone computer-mediated intervention (no web-based or mobile-device based component)
Outcome	Recovery Self-management Illness management	Interventions that aim to support personal recovery and/or self-management	Interventions that focus solely on clinical recovery such as symptom reduction or treatment compliance

**Fig. 1:**
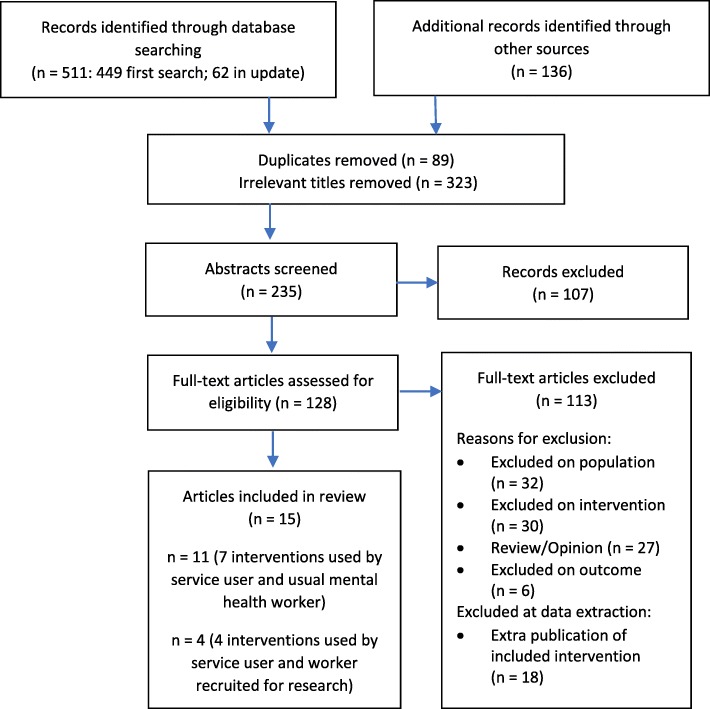
Flow chart of study selection process

Additional searching included tracking citations in reviews that were found in the searches but did not meet the inclusion criteria, and in the included studies; a Google Scholar alert (running to Dec 31, 2017); and a grey literature search of ProQuest Dissertations, Theses Global, SCOPUS Proceedings, BMC Proceedings and the Grey Literature Report. Key word searches were conducted in six relevant journal titles including the Journal of Medical Internet Research Mental Health and Psychiatric Services. Finally, database searches in Medline, CINAHL and Embase were updated to December 2017. A total of 558 citations, excluding duplicates, were identified. Figure 1 outlines the study selection process.

#### Stage 3: Study selection

The first author reviewed titles and abstracts against inclusion and exclusion criteria that were iteratively refined as recommended [26, 27], in consultation with authors two and four. The second author independently reviewed 10% of randomly selected titles and abstracts. High levels of agreement were recorded on title (93%) and abstract (80%) decisions and any disagreements were resolved by consensus. As shown in Fig. 1, 323 irrelevant titles were removed when it was clear from the title that the study did not involve an Internet-based intervention, or participants who experienced psychosis, or both. A further 107 studies were excluded after reading the abstract clarified that they also did not meet the inclusion criteria.

We reviewed the full text of the remaining 128 studies (Fig. 1). The first and second authors met regularly during this phase to review papers and to reach consensus on studies that met the inclusion criteria. Thirty-two studies were excluded at the full text stage, as on review the population did not clearly include people who experienced SMI. We defined jointly using an Internet-based intervention as occurring when the pair engaged with the intervention, including communicating through and/or about the intervention, whether synchronously or asynchronously. Thirty studies were excluded as not meeting the intervention inclusion criteria. Examples included workers interacting with an online community, rather than having one-to-one working relationships with service users (e.g. [30]), and computer-mediated interventions without a web-based or mobile device-based component, such as electronic decision support systems [31, 32]. We included studies of Internet-based shared decision making (SDM) tools, given that sharing decisions about treatment requires a partnership between a service user and provider and supports the process of “recovering a life after a diagnosis of a major mental disorder” [33]. Other studies were excluded if they were review or opinion articles not directly relevant to the population and intervention, if outcomes addressed in the study were clinical, such as treatment compliance, or when there were multiple studies about the same intervention and population that did not add further information to studies that were already included (Fig. 1).

Fifteen studies remained after we applied the clearly defined inclusion criteria. We distinguished between primary studies (*n* = 11), in which an Internet-based intervention was used within an existing service user-provider working relationship, and secondary studies (*n* = 4), where research workers were employed to provide the intervention.

#### Stage 4: Charting the data

The quality of included studies was appraised to support the dissemination of useful scoping review findings [28]. The first author appraised the included studies using the Mixed Methods Appraisal Tool (MMAT-v2011) [34, 35], an efficient tool to appraise diverse study designs [36, 37]. Each study appraisal was converted to an overall quality score from low (0) to high (4 points). Second, to answer review questions 1 and 3, the first author extracted descriptive data and relevant findings. The Template for Intervention Description and Replication (TIDieR checklist) [38] was used to document each intervention. Extra publications describing the development of the interventions were consulted to add further detail. An additional text file provides this information (Additional file 1). The relatively small number of included studies and regular reviewer consultation during this phase negated the need for independent data extraction.

#### Stage 5: Collating, summarizing and reporting results

Next, extracted data were collated and summarized as suggested [25, 26] into a descriptive and narrative summary of study characteristics that addressed the first and third review questions. Three specific recovery-oriented working practices were considered to answer the third question: understanding service users’ values; assessing and amplifying their strengths; and supporting goal striving [23, 39]. The first author completed a qualitative data-driven thematic analysis [40] to answer the second review question. Qualitative findings related to interactions between service users and mental health workers when using an Internet-based intervention were coded in qualitative software as recommended by Levac et al. [26], using QSR’s International NVivo 11 data analysis software [41]. The coding process was recorded in a memo and reviewed with the third author. All authors contributed to reporting the summarized results.

## Results

The results are presented in three sections: first a summary of characteristics and quality of the included studies is provided. Second, to answer the first review question, the Internet-based interventions that were the focus of these studies are outlined, presented separately for interventions used with a usual mental health worker and those used with a research worker. The third section considers the influence of jointly using an Internet-based intervention on mental health practice to answer research questions two and three.

### Summary of included studies

#### Study characteristics

The 15 included papers outlined 11 Internet-based interventions used in mental health services in Europe (5 interventions), North America (5 interventions) and Australia (1 intervention) by service users and mental health workers. Seven interventions engaged service users and their usual mental health workers [42–52] (Table 2). Of these, two publications were included for the Mieli.Net [42, 43], Mental Health Engagement Network (MHEN) [45, 46], CommonGround [47, 48] and ReConnect [51, 52] interventions. Four interventions engaged experienced mental health workers employed in a research project [53–56] (Table 3). The mental health workers were most commonly psychiatric nurses in community health settings, however social workers, occupational therapists, psychologists, psychiatrists and peer workers also participated. All interventions included web-based elements such as websites, online forums and Internet diaries. Five also incorporated mobile-device based interventions in the form of native smartphone apps [45, 49, 53, 55, 56]. People with lived experience of mental illness were described as having explicit researcher roles in the Momentum [49] and ReConnect [52] studies only.

**Table 2:** Study characteristics: Interventions used with usual mental health worker

Study	Design/MMAT Rating	Setting/Country/Year	Intervention	Service users/ diagnosis	Workers/ profession
Anttila, Koivunen [42]	Qualitative description: worker questionnaires after 12 months using intervention 2/4	Two inpatient psychiatric hospitals, Norway, 2005-2006	*Mieli.Net* WB: patient education portal to support SM. Includes information, peer and staff support channels. Nurses used portal to deliver 6 education sessions over 1 month. SU continued access post discharge.	*n* = undisclosed Available to inpatients diagnosed with schizophrenia	*n* = 56 psychiatric nurses participating in study
Koivunen, Huhtasalo [43]	Qualitative description: worker FGs and 1:1 IVs after providing systematic IT education 2/4				*n* = 30 (subset of above). 14 nurses provided IT based education and 16 provided conventional education
de Leeuw, van Splunteren [44]	Mixed methods^a^ Single group pilot study: qualitative results only. SU and worker open-ended questions at BL and after 9 months; and FGs at 15 months. 0/4	Two MH organizations, Netherlands, 2009-2011	*Personal Control in Rehabilitation (PCR)* WB: SM & communication portals including information, SM, communication tools. SU could authorize worker and carer access.	*n* = 19 (FG participants); 100% schizophrenia or first episode psychosis; 74% male; aged16-66 years	*n* = 36 15 nurses, 8 psychiatric nurses, 4 SW, 3 psychologists, 3 psychiatrists, 3 managers
Forchuk, Rudnick [45]	Mixed methods, Initial analysis of data from two group, delayed intervention RCT. SU questionnaire at BL, 6, 12, 18 months and usage data. SU and worker FGs held over 18 months 0/4	Four community MH agencies, Canada, 2012-2014	*Mental health engagement network (MHEN)*WB and MDB: App with personal health record and SM tools. Smart phone provided to SU; tablet device provided to workers for both to access health record.	*n* = 400 59% Psychotic disorder; 60% male; mean age 37 years	*n* = 54 Nurses, SW, OT
Forchuk, Donelle [46]	Mixed methods, SU questionnaires, and FG data as illustrative quotes 1/4			*n* = 394 (same sample as above)	
Goscha and Rapp [47]	Qualitative description: 2 x 1:1 SU and worker IVs after 4 months intervention use 3/4	One community MH centre, Kansas, USA 2006-2007	*CommonGround* WB: SDM program with peer content and peer support, used to create health report. Report viewed by prescriber and used in appointments to make shared decisions, final plan shared with treating team.	*n* = 12 SMI: Unspecified % schizophrenia/psychotic disorders; 58% male; mean age 45 years	*n* = 15 5 CM, 3 prescribers, 3 nurses, 2 PW, 2 supervisors
Bonfils, Dreison [48]	Mixed-methods: worker 1:1 IV at end of intervention use, SU usage data, fidelity reports 2/4	One urban community MH centre, Indiana, USA 2013-2015		*n* = 167 SMI: 67% schizophrenia diagnosis; 57% male; age not disclosed	*n* = 12 supervisors, PWs, psychiatrists, managers
Korsbek and Tonder [49]	Qualitative description, single group pilot study: worker FGs, SU 1:1 IVs held after using intervention 4 months 1/4	Hospital, community MH, psychosis treatment centre, Denmark, Year not stated	*Momentum* MDB and WB: SDM app, with peer support. SU used to prepare for meeting and could authorize worker access. Workers logged in to treatment site to view shared preparations.	*n* = 7 of 78 participants with SMI, including schizophrenia, affective disorders; gender and age not disclosed	*n* = 19 12 workers: nurses, OT, psychologist, SW; 7 doctors
Blankers, van Emmerik [50]	Quantitative non-randomized, two group pilot study: blended FACT (with SM focus) and conventional FACT Standardized SU questionnaires at baseline and 3 months 2/4	SMI community treatment centre, Netherlands, 2012-2013	*Blended flexible assertive community treatment (Blended FACT)* WB: Information and education portal, appointment scheduling and a peer forum. Skype contact with nurses. Computer, Internet and webcam provided to SU.	*n* = 47 SMI including 40% schizoaffective disorder or schizophrenia; 47% male; mean age 46 years	*n* = undisclosed psychiatric nurses
Gammon, Strand [51]	Mixed-methods: single group (two sites) exploratory study. SU questionnaires BL; usage data; SU and worker FGs after using intervention 3 months; SU discussion groups and forum posts; documents 2/4	Multiple health services, 1 urban, and 1 rural community, Norway, 2015-2016	*ReConnect* WB: recovery-focused portal with resources and information, messaging with providers, peer support forum; workers had partial portal access.	*n* = 29 Receiving MH services at least 6 months; including 10% schizophrenia-related diagnosis; 14% male; mean age 44 years	*n* = 27 including 11 nurses, 5 SW, 3 physicians
Strand, Gammon [52]	Qualitative description: SU and worker FGs early stage; 1:1 IVs and 1 dyad IV late stage 4/4			*n* = 14 (subset of above)	*n* = 17 (subset of above)

*BL* baseline, *CM* case manager, *FG* focus group, *IT* information technology, *IV* interview, *MDB* mobile-device based, *MH* mental health, *OT* occupational therapist, *PW* peer worker, *RCT* randomized controlled trial, *SDM* shared decision making, *SM* self-management, *SMI* severe mental illness, *SU* service user, *SW* social worker, *WB* web-based ^a^MMAT rating for qualitative study only as no quantitative results reported

**Table 3:** Study characteristics: Interventions used with worker employed in research

Study	Design/MMAT Rating	Setting/Country/Year	Intervention	Service users/ diagnosis	Workers/ profession
Baumel, Correll (53)	Quantitative, single group, descriptive study: contact logs and SU survey after 6 months of use 1/4	Ten MH services, USA, 2013-2014	*Health Technology Program (HTP)* WB and MDB: smartphone illness SM app, website with daily support and education resources. Smartphone and laptop provided to SU. Workers support use, view website data and respond to messages.	*n* = 200 schizophrenia, schizoaffective disorder, psychotic disorder currently or recently in hospital; 59% male; mean age 34 years	*n* = undisclosed Health technology coach (research role): trained CM
Thomas, Farhall (54)	Quantitative, feasibility pilot, single group study after 3 months use: SU questionnaires, usage data, and post intervention 1:1 IV used as illustrative quotes 4/4	Community MH service, Australia, 2015	*Self-Management and Recovery Technology (SMART)* WB SM and recovery-focused website. Tablet device provided to worker to use website with SU in 8 sessions.	*n* = 10 psychotic disorder; 90% male; mean age 42 years	*n* = 2 MH worker (research role)
Schlosser, Campellone (55)	Quantitative, single group feasibility study in intervention design phase and prior to RCT: SU satisfaction and usage data, after 3 months use. 1:1 IV used as illustrative quotes 4/4	Early psychosis clinic and community treatment centres, USA, year not stated	*Personalized Real-Time Intervention for Motivation Enhancement (PRIME)* MDB and WB: smartphone app for SU to select and monitor goals, communicate with peers, and worker. Profile viewed by worker on website. Smart phone provided to SU.	*n* = 20 recent onset schizophrenia (10 used PRIME version 1, 10 used PRIME version 2); 85% male, mean age 23 years	*n* = 6 Motivation coaches (research role): Masters-level clinicians
Fortuna, Dimilia (56)	Quantitative, pilot feasibility study, single group: SU questionnaires at BL, 1 and 3 months; fidelity assessment; usage data 3/4	Community clinical teams, USA, year not stated	*PeerTECH* MDB and WB: smartphone SM app, WB eModules delivered in sessions with PW, text messaging with PW. Smartphone provided to SU.	*n* = 8 Older adults, SMI and co-morbid chronic medical conditions: 25% schizophrenia diagnosis; 12.5% male; mean age 68 years	*n* = 3 Certified peer specialists (research role)

*BL* baseline, *CM* case manager, *IV* interview, *MDB* mobile-device based, *MH* mental health, *PW* peer worker, *RCT* randomized controlled trial, *SM* self-management, *SMI* severe mental illness, *SU* service user, *WB* web-based

#### Study quality

All studies were exploratory in nature, including five qualitative, five mixed-methods and five quantitative studies (Tables 2 and 3). Methodological quality using the MMAT-v2011 tool [35] varied considerably. Qualitative studies were rated from 0 to 4 points, quantitative from 1 to 4 points and mixed methods studies from 0 to 2 points. The mixed methods ratings were lower as their scores are determined by the lowest score of either the qualitative or quantitative component [35].

Descriptions of study quality based on the MMAT-v2011 can provide more useful information than the scores, given that the quality of reporting different study designs varies [35]. A strength of the qualitative studies and qualitative components of mixed methods studies was sourcing information-rich participants’ experiences. However, qualitative data from both service users and workers were collected in less than half (40%) [44, 45, 47–49, 51, 52] of the 15 studies. Ratings for the qualitative studies were also limited by researchers minimally considering their influence on the qualitative findings, other than in ReConnect [52]. The quantitative studies and quantitative components of mixed methods studies reported predominantly descriptive data on use of and satisfaction with the interventions. This included feasibility and acceptability research for a sample of 200 participants (HTP) [53], and three small samples (n ≤ 20) (PeerTECH, SMART, PRIME) [54–56]. There was also one mixed methods study with 400 service user and 54 worker participants [45]. Using the MMAT-v2011 [35] criteria, a strength was that quantitative measures used in the studies were usually described clearly and standardized (for example, to measure quality of life or empowerment). Ratings were reduced by missing information about the study sample, such as justification of the sample size or why eligible participants chose not to participate.

### Outline of Internet-based interventions

Key details of the intervention goals, tools and use are summarised below, to provide an outline of the types of Internet-based interventions that were the focus of each study. Table 4 and Additional file 1 provide further details to support the following narrative account.

**Table 4:** Intervention details

Internet-Based Intervention Feature		Usual MHW							MHW employed for research project			
		Miele.net, Finland [42, 43]	PCR, Netherlands [44]	MHEN, Canada [45, 46]	CommonGround, USA^a^ [47, 48]	Momentum, Denmark [49]	Blended FACT, Netherlands [50]	ReConnect, Norway [51, 52]	HTP, USA [53]	SMART, Australia [54]	PRIME, USA [55]	PeerTECH, USA [56]
Purpose	Self-management	✔	✔	✔			✔		✔	✔		✔
	Recovery				✔	✔		✔		✔	✔	✔
	Enhance communication / SDM		✔	✔	✔	✔	✔	✔				
Training and support	Training/support provided to SU	✔^b^	✔	✔	✔	✔	✔	✔	✔	✔	✔	
	Training provided to workers	✔	✔	✔	✔	✔		✔	✔	✔	✔	✔
Technology components	Internet-based information portal	✔	✔		✔		✔	✔	✔	✔		✔
	Smartphone / tablet computer app			✔		✔			✔	✔	✔	✔
	Tools to record own content		✔	✔	✔	✔		✔	✔	✔	✔	✔
	Information/tools to support daily living	✔		✔	✔	✔	✔	✔	✔	✔	✔	
	Communication channel with worker	✔^c^	✔	✔		✔	✔	✔	✔		✔	
	Peer communication – digital or F2F	✔	✔		✔		✔	✔		✔	✔	✔
	Peer narratives (e.g. photos, videos, audio)	✔			✔					✔	✔	✔
Worker, SU interactions	Face-to-face using technology - prescribed	✔			✔				✔^d^	✔	✔^d^	✔
	Face-to-face using technology – flexible				✔	✔		✔	✔			
	Internet-based – prescribed										✔	
	Internet-based – flexible		✔	✔		✔	✔	✔	✔		✔	✔
Reported Benefits	Increases SU autonomy – worker view	✔	✔	✔		✔		✔				
	Expands conversations – worker view	✔			✔	✔						
	Facilitates goal-related communication – SU view		✔		✔	✔		✔				
Reported Barriers	Difficulties logging in	✔		✔	✔	✔	✔	✔				
	Extra workload, not integrated in service system	✔	✔	✔	✔	✔		✔				
	Unsuitable for SU – worker view	✔			✔	✔		✔				

*F2F* face-to-face, *MHW* mental health worker, *SDM* shared decision making, *SU* service user a. CommonGround Indiana and Kansas combined b. Training provided by worker in first session; c. Online Q&A column, not 1:1 communication with specific worker; d: First meeting to prepare for using intervention, incorporating digital tools

#### Interventions used with usual mental health workers

##### Intervention goals

The primary goal of the seven interventions was to facilitate service user self-management [42, 44, 45, 50]; promote shared decision making [49]; and to support personal recovery via increasing service user involvement in care and collaboration with providers [47, 51]. Authors referenced concepts including self-efficacy [42], self-determination [47], and recovery-oriented practice [49, 51], with a focus on the need for interventions that facilitate service users’ active role in managing their health.

##### Intervention tools

Two tools were present in 6/7 interventions: a communication channel with workers and information or tools to support daily living. Communication occurred digitally through the Internet using email, text or video chat [44, 45, 50, 51]. Sharing information to support daily living was facilitated by workers being able to view service user generated digital content, such as their preparation for meetings [49], mood monitoring [45], and personal goal statements (e.g.[47, 51]). In Miele.net [42, 43] and ReConnect [51, 52], workers and service users could also access and discuss online information or tools together in face-to-face meetings. Service user communication with peers through a forum or text messaging was also possible in four interventions [42, 44, 50, 51], while CommonGround [47, 48] and Momentum [49] facilitated direct contact with a peer specialist.

##### Intervention use

The interventions enabled flexible Internet-based communication between service users and workers, often associated with preparing for their meetings (e.g. [42, 47–50]). Service users could also use information websites, online health records and other online tools when they chose (e.g. [42, 44, 46, 51]). Uptake of the interventions was varied. For example, in ReConnect [51] workers’ website use ranged from never to almost daily. In another example of varied use, only 65% of service users completed a web-based health report at least once over a 20-month period in the CommonGround (Indiana) study [48]. Intervention use also diminished over time in the longer duration studies. Site visits to the PCR website initially dropped and then stabilized in months 5 and 6 [44]. In the MHEN study, 93% of service users were using their smartphone after 12 months, but only 45% were still accessing their web-based health record [46].

Factors that contributed to low use included complex logging-in procedures (6/7 interventions), the intervention not integrating with other Internet-based systems in the service (6/7 interventions), and workers believing that service users were not suited to using the intervention (4/7 interventions) (Table 4). Some service users were deemed unsuitable by some workers on the grounds of being too sick [42], too low functioning [49] or likely to be intimidated by using computers [48]. In contrast, intervention use was facilitated if the pre-existing relationship between a worker and service user was positive [47, 49, 51], if workers encouraged and supported service users with the intervention [49], and if expectations about how they would both engage with the intervention were clarified [44, 52]. Worker engagement with the intervention was “considered essential for successful use by some consumers” [52].

#### Interventions used with research workers

##### Intervention goals

Two of the four Internet-based interventions used with mental health workers employed for a research project aimed to support self-management, one with a focus on relapse prevention for people recently discharged from hospital care for schizophrenia [53] and one addressing self-management of mental and physical health in older people [56] (Table 4). SMART aimed to promote personal recovery and self-management [54]. PRIME aimed to improve motivation and functioning in people with recent onset schizophrenia and was included as the approach incorporated self-determined goals and “a collaborative, reinforcing and strengths-based approach” consistent with personal recovery [55].

##### Intervention tools

All were multi-component smartphone-accessible interventions. Smartphones were provided to service users if needed in three studies [53, 55, 56], while SMART workers used a tablet computer in their meetings with service users [54]. Tools embedded in the interventions included educational modules (3/4 interventions); peer communication tools (3/4); and peer videos (2/4).

##### Intervention use

Adherence to interventions appeared to be overall high. The research worker’s role included having face-to-face meetings with participants at the set-up stage only [55], regularly over three months [54, 56], or for six months [53]. This prescribed face-to-face contact contrasted with the interventions used with usual mental health workers, where it was less common for workers and service users to use the interventions together face-to-face (Table 4). All interventions excepting SMART also enabled text-based chat or messaging between workers and individual participants. Usage data indicated that all four interventions were feasible and acceptable.

### Influences of jointly using an Internet-based intervention on practice

#### Influences on interactions between service users and workers

Key findings about the influence of Internet-based interventions on interactions between service users and their usual workers were synthesized into two preliminary themes: “working together” and “feelings of mistrust and not being appreciated”. Related findings from the studies with research workers are included in these themes, where relevant.

##### Working together

Collaboratively using an Internet-based intervention had the potential to create a *“sense of working together”* [43] for service users and their usual mental health workers. Working together arose when the Internet tools and communication channels opened conversations between service users and workers [42, 47–49, 52], as indicated by a nurse using Mieli.Net:

*“There have been good conversations in education sessions, so I consider web pages to be quite helpful in nursing.”* ([42], p.151).

These conversations were described by workers as “productive” [48], “wide-ranging” [42] and about what was important to service users [52]. Service users reported that access to online tools, information and messaging enabled them to write down their ideas and to ask questions when they wanted rather than waiting for their next appointment [44, 52]. They appreciated their workers being more available [45] and shared more information:

*“They wouldn’t get as much information out of me (prior to CommonGround)…once I tell the computer what my situation is, and they discuss it with me…that has helped”* ([47], p.270).

Working together was further enhanced when service users reflected on and communicated their goals through the intervention [44, 45, 47, 51]. Primarily, meaningful service user goals became visible, injecting a specific direction to working together. Workers perceived that service users could *“set an agenda of their own and structure what was important”* [49]*.* Workers could then support personal goal striving, as indicated by this worker in ReConnect:

*“The goal module has really helped. When he/she says, “I wish I’d do more of this,” then I can put pressure on. When it’s written down in there as a concrete goal, then it kind of lights up a fire of sorts.”* ([51], p.8).

Service users perceived that they were more in control of decision making [44, 45, 47, 52], as discussions gained new starting points, a clear focus and incorporated their resources and goals. In the MHEN study, the authors asserted that:

*“Since adopting this technology, many clients expressed feelings of greater control in their health management and in their life”* ([45], p.4).

Pre-existing working relationships influenced whether working together occurred [49, 51]. For example, existing trusting relationships overcame service users’ initial fears about using the intervention [49]. In ReConnect, the researchers considered that pre-existing poor working relationships were exposed when workers did not use the intervention with service users [51]. Engagement with the intervention, required to facilitate working together, was supported when workers [42], service users [46] or both [49], perceived the intervention to be easy to use.

Consistent with the above analysis, participant quotes from the SMART study suggested that interesting discussions were elicited and guided through using a website with a worker employed for a research project. One SMART participant noted that discussions were less intimidating and more directed towards *“what you had to say and what you thought about situations*”. Another SMART participant stated:

*[Without the website] “we wouldn’t have had nearly as much to talk about. And then I would have been more stuck for words I think. I wouldn’t have been able to talk about all the issues that we had discussed about the website”* ([54], p.8).

In the PRIME study involving research workers as coaches [55], it was noted when coaches sent frequent, brief, and casual messages to participants, service user responses changed from a ratio of 12:1 (coach initiated interactions to service user response), to a ratio of 3:1. In summary, these studies demonstrated that using the intervention with a research worker supported regular communication between the user and worker on a range of topics. Factors that supported this outcome were hypothesised to include the opportunity for daily messaging [55, 56] and workers providing training and technical assistance to service users [53].

##### Feelings of mistrust and not being appreciated

In contrast, relationships could be negatively affected by *“feelings of mistrust and not being appreciated”* [52], that arose when expectations about getting started with the intervention or the frequency of each party’s engagement were unreciprocated or mismatched [44, 45, 48, 51]. This was particularly so for service users in ReConnect when their workers failed to actively participate in the Internet-based intervention:

*“Why did [provider] agree to work with me through this tool if she never expected to do it? She should have just said no. You get so disappointed”* ([51], p.9).

Workers could also experience frustration if they perceived that a service user was hard to engage in the intervention, as described by a nurse in Miele.net:

*“It’s hard work on busy days with an uninterested patient”* ([42], p.151).

Influences on workers’ decisions to actively use the intervention included some workers being concerned not to burden service users [51], or them assuming service users were too sick [42], or too poorly functioning [49] to use the intervention. Workers also forgot to look for online messages, or often had difficulty fitting the intervention into their workload and existing systems [42, 44, 45, 52]. A worker in CommonGround Indiana noted:

*“I feel like they (workers) have so much they have to do already that trying to say, “hey, make sure you get people in for CommonGround” would just feel overwhelming to them”* ([48], p.4).

Some ReConnect workers described waiting for service users to initiate use of the intervention, as they saw this as being consistent with their clients taking more control [52]. However, some service users were uncertain what they needed [52] or did not want to disturb their worker [49, 52]. In Momentum it was noted that service user initiation could be stalled if there was an imbalance of power in the relationship:

*“a consumer explained that she did not dare to share it (her treatment preparation), as some of her considerations might be irrelevant for the staff”* ([49], p.171).

In addition to the above barriers, workers in ReConnect could find it difficult to interact with service users through the Internet due to worrying that their text messages might be misinterpreted or feeling overwhelmed by long and frequent messages from service users [52].

#### Influences on recovery-oriented practice

Results for this section consider all 11 interventions. Recovery-oriented practices as outlined by Slade et al. [39] appeared to be elicited when Internet-based interventions were used by service users and workers in mental health services. Firstly, service users’ values and treatment preferences were identified and integrated into work with mental health workers. This was demonstrated through workers noting that using the intervention enabled service users to set the agenda for appointments [49, 52], and gave them more control of treatment decisions [44, 45, 47, 49, 52]. Characteristics of the interventions including information portals (e.g. [42, 44]) and interactive tools ([e.g. [52, 54]) supported service users to communicate about topics that mattered to them. Secondly, functions included in several interventions supported user-generated goal striving, for example, the goals and activities in ReConnect [51]; the power statement in CommonGround [47, 48]; and the daily challenge in PRIME [55]. While the practice of assessing and amplifying service user strengths was less explicitly evident, two studies identified that using the intervention helped workers to be more aware of service users’ resources [42, 51], and exercises related to identifying strengths and resources were included in ReConnect [51] and SMART [54]. Recovery-oriented practices were less evident when the intervention was not well integrated into practice or not used by both parties.

## Discussion

### Principal findings

This review substantiates that Internet-based interventions designed for joint use by people experiencing SMIs and mental health workers, with a focus on self-management, recovery and shared decision-making, are increasingly available in mental health services. Nine of the 15 included studies (60%) were published since 2016 and only three of the 11 identified interventions were included in a previous related review [24], demonstrating the growing pace at which jointly used Internet-based interventions are developing. These facilitated service users’ active contributions towards their health and care planning and increased their opportunities to communicate with workers and peers about what was important to them. Furthermore, when well-integrated into practice with strong worker engagement, these Internet-based interventions demonstrated potential to positively influence interactions between service users and workers and to promote recovery-oriented practice. They increased focus on service users’ goals and treatment preferences. Although the quantity and quality of evidence available is modest, it suggests that joint use of Internet-based interventions between service users and workers can bring a person-centred and goal-oriented focus to discussions. However, the review also establishes that Internet-based interventions can negatively influence interactions between service users and workers and suggests that they will not necessarily change practice if the pre-existing working relationship is poor.

### Key features of Internet-based interventions that supported their joint use

Recommendations that Internet-based and mobile interventions need to be flexible and developed with user input to cater for a range of users experiencing SMI [17] were evident across the interventions in this review. Service-user and worker consultation was common in intervention design, reflecting a user-centred approach that is essential to creating engaging and easy to use interventions [17] and supports innovation success in mental health services [57, 58]. Training and support were also frequently offered to service users and workers in the 11 interventions, factors that are conducive to success [57]. However, there is scope for greater participation of service users in research related to these interventions, as has been possible in other areas of recovery-related research [59, 60].

Mobile technologies, specifically smartphone apps, may be particularly important to include, as people experiencing SMI feel comfortable using their own devices; apps can support remembering, initiating and maintaining strategies [21]; and smartphone interventions are quicker to use [18, 20]. This was demonstrated by service users in the MHEN study preferring to use smartphone applications for appointment and medication reminders rather than their web-based health record [46]. The interventions with web-based portals (e.g. [42, 51]) were also valuable, as these provided service users and workers access to a wide range of information and tools. In several interventions, providing smartphones [45, 55, 56] or computers to service users [50, 53], and tablets to workers [46, 54], supported intervention engagement. Although rates of mobile phone ownership are high and increasing among people experiencing psychosis [10, 11], a small group of older people continue to experience digital exclusion [21, 61] and may benefit from devices being provided.

It is notable that 9/11 interventions included contact with peers, as digital communication in six interventions and as face-to-face support in five. Opportunities for online communication with peers has potential to increase service users’ hope [62, 63]. Further research investigating the role of peers in Internet-based interventions used with workers is warranted, as hope has previously been identified as a missing theme in Internet-based self-management interventions [19].

### Influence of jointly using Internet-based interventions on interactions

Preliminary evidence suggests that Internet-based interventions used by service users and workers can create a positive sense of working together. Digital tools for communication and shared decision-making that facilitated service users to explore, record and discuss their health, goals, treatment preferences and care plans (e.g. [48, 51, 54]) appeared to contribute to this outcome. When integrated into the working relationship, using Internet-based interventions could lead to more productive discussions (e.g. [42, 47, 49, 51, 54]) and service users gaining more control over health-related goals and decisions (e.g. [44, 45]). In comparison, not working in partnership may be indicated when there is no meeting agenda and agreement about the primary purpose of psychiatric visits is low [64]. Likewise, care plans can be perceived as being only relevant to professionals and inconsequential to everyday life when service users are not partners in their development [65]. While intervention component use could diminish over time (e.g. [46]), the ongoing benefits of accessible communication (e.g. [44, 45, 55]) and a stronger link to service user goals in meetings (e.g. [48])] could support continued use.

Working relationships were not always strengthened when service users and workers used Internet-based interventions. Mismatched expectations about initial and subsequent use could result in frustration and disappointment, negatively influencing the trust that characterizes therapeutic relationships between service users who experience SMI and mental health workers [66]. For example, trust may have been broken if a service user perceived that the worker lacked commitment, as demonstrated by them not investing enough time to attend to the Internet-based modules or messages that they posted. Additionally, when workers made decisions about which clients were suited to using the intervention, this suggested a paternalistic approach, more aligned to clinically defined than personally defined recovery [67].

Our review indicates that mental health workers’ engagement in Internet-based interventions is an important factor in engaging service users experiencing SMI. Previous research has identified that workers have varying views about using Internet-based resources with service users, which include workers anticipating taking more, or less control of the resources [68]. This suggests that expectations about using Internet-based interventions need to be clarified between workers and service users. Agreements could be made about who will initiate use, the frequency of use and how the intervention will be used in face-to-face meetings, as well as online. As also identified by Strand, Gammon [24], workers’ engagement was affected when Internet-based interventions duplicated other processes, were difficult to fit into work flows or overwhelmed workers. Fitting Internet-based interventions into work flows is a common challenge in health settings [69]. This finding supports the call for close consideration of organizational factors in addition to stakeholder consultation during the initial phase of creating technology-enabled services [58] in mental health.

### Opportunities to support recovery-oriented practice through jointly using Internet-based interventions

Using Internet-based interventions offers potential to embed aspects of recovery-oriented practice into working relationships. Identifying this potential is important, because the nature of relationships matters in recovery-oriented practice [3, 4, 70]. Internet-based interventions demonstrated potential to elicit service user’s values and treatment preferences and support their goal striving (e.g. [47, 55]), two working practices targeted for recovery-oriented practice [39]. This is consistent with service users reporting that it is helpful for their recovery when services support them to achieve self-identified life goals [71]. Sharing this information with workers set the agenda for meetings and increased service user involvement [49, 52], promoting a partnership-style of relationship, as supports recovery [3]. The next generation of Internet-based interventions could include more resources that focus on service-user strengths [72], to further support recovery-oriented practice.

### Future research

Several research design issues are evident from this scoping review. Firstly, rigorous quantitative studies are still needed to demonstrate intervention effectiveness, as most evidence to date is exploratory and descriptive. Another important issue is the need for Internet-based interventions to become sustainable within mental health services [58]. Arguably, sustainability will be easier if usual mental health workers are involved in future intervention studies, rather than research workers as occurred in four of the interventions included in this review. Researchers completing a detailed eHealth checklist [73], including specifying the theoretical framework when reporting interventions will support greater intervention transparency and more specific detail than can be reported using the TIDieR checklist [38]. The nature and quality of support influences helping relationships [22, 23, 70, 74], so understanding more about the type of support needed by service users when using Internet interventions with their worker is warranted in future research. Dyad interviews could facilitate exploring the shared experience and meaning of using Internet-based resources together [75] and build knowledge about how dyads negotiate expectations and the worker actions that support intervention use. Finally, as this field of research matures, international research collaborations would be beneficial to increase access to Internet-based interventions in more mental health services across the world. Collaborations could also reduce the inherent risk of rapid obsolescence in technology-enabled services [58] if many research teams with time-limited funding continue to develop new Internet-interventions.

### Strengths and Limitations

A comprehensive and iterative search phase was a strength of this review. However, strict inclusion and exclusion criteria meant that several Internet-based interventions for people experiencing SMI designed for use in mental health services were excluded. For instance, an intervention like HORYZON [30] included elements that could be used by service users and workers together, although this type of use was not reported in the published research. Including studies where a research worker, rather than a usual mental health worker, engaged with a service user could reduce applicability to usual working relationships. These studies were considered separately when exploring the impact of using Internet-based interventions on service-user and worker interactions, to limit this risk. The depth of qualitative data was variable in the included studies, with only one study focusing specifically on working relationships [52]. Further high-quality qualitative research is needed to expand the preliminary findings regarding working together that were synthesized from existing mixed methods and descriptive qualitative research. Finally, consultation with stakeholders as recommended in revisions to scoping review methodology [26, 27] was not included, primarily because the field of research is young, and timely and broad consultation was not feasible. Assuming the pace of development continues, this will be an important factor to address in future reviews.

## Conclusions

Rapid growth in Internet-based interventions for people experiencing SMI has recently translated into more readily available Internet-based interventions focused on self-management and recovery for service users and mental health workers to use together in mental health services. The findings from this scoping review indicate that when these Internet-based interventions are well-integrated into practice, they can support service users’ involvement in their care, promote a sense of working in partnership, and support recovery-oriented practices. However, mistrust in service user-provider working relationships can also develop if expectations about using the intervention are not clarified, and if the interventions are not embedded into usual mental health service systems. The challenge for future practitioners and researchers alike is to attend to the human support component of Internet-based interventions with the same level of attention that has been given to developing and implementing their technological components.

## Additional file

Additional file 1: Jointly used Internet-based interventions - TIDieR intervention details and study findings. (DOCX 80 kb)

## Data Availability

Not applicable

## Abbreviations

CINAHL: Cumulative index to nursing and allied health literature
FACT: Flexible assertive community treatment
HTP: Health technology program
MHEN: Mental health engagement network
MMAT: mixed methods appraisal tool
PCR: Personal control in rehabilitation
PRIME: Personalized real-time intervention for motivation
SDM: shared decision making
SMART: Self-management and recovery technology
SMI: Severe mental illness
TIDieR: Template for intervention description and replication
