# Supplementary material for: Internet-based interventions to support recovery and self-management: A scoping review of their use by mental health service users and providers together
Source: BMC Psychiatry. 2019 Jun 20;19:191. doi: 10.1186/s12888-019-2153-0 (PMC6585058; doi:10.1186/s12888-019-2153-0)
Supplement: Supplementary file 1 — Jointly used Internet-based interventions - TIDieR intervention details and study findings. (DOCX 80 kb) [file 12888_2019_2153_MOESM1_ESM.docx]

| 1. INTERNET-BASED INTERVENTIONS USED WITH USUAL MENTAL HEALTH WORKER | | | | | | |
| --- | --- | --- | --- | --- | --- | --- |
| INTERVENTION/  WHY  TIDieR Q1,2 | MATERIALS  TIDieR Q3 | PROCEDURES & PROVIDER  TIDieR Q4,5 | DELIVERY: HOW, WHERE, WHEN  TIDieR Q6-8 | TAILORING, ADHERENCE  TIDieR Q9-12 | KEY FINDINGS RELATED TO INTERVENTION USE BY SERVICE USER AND WORKER |  |
| ***Mieli.net***  *Goal:* To support patient self-management skills:   - Develop problem-solving skills - Improve self-efficacy - Support knowledge for life situations, using access to evidence-based information   *Theory:* Self-management: to develop problem-solving skills, improve self-efficacy and support knowledge in real-life situations. | (Välimäki et al., 2008)   - Patient education delivered using a website - Sessions structured around: illness, treatment, wellbeing, patients’ rights and peer support, and daily life and eSupport tools - Website included evidence-based, multimedia information, including stories (voices, photos) from other patients and links to web-pages; internet-based peer support; eSupport tool for counselling and support from staff | - Nurses IT skills assessed, then trained to use website and provided with manual - SU introduced to portal and IT skills assessed, then sessions using portal commenced - Help available from researchers if needed - Workers were psychiatric nurses   (n = 56, mean age 41 years) | - Nurses delivered one introduction session and five x 1:1 sessions with average length of 40 mins, over 1 month in 2006 - Nurses and SU looked together for information on the closed portal to answer SU questions - Sessions delivered on the ward, two acute hospitals, Finland - SU had autonomous access to portal outside of education sessions, post-discharge - SU could also interact with peers in discussion room and post question for staff to answer on Q&A board | As well as the standardized topics, participants could explore self-selected information and nurses could tailor information and IT training to participant needs | (Anttila, Koivunen, & Välimäki, 2008) Qualitative data from workers  *Advantages of IT-based standardized education for SU:*   - Source of information: an effective and fast way for SU to obtain information - Self-help aid: encouraged SU to ask questions, supported conversations between patients and nurses, including on “sensitive topics that were difficult to talk about” when SU “mental state was good” (p.150) - Usability: website was easy to use for SU with computer skills   *Advantages of IT-based standardized education for workers (nurses):*   - Nursing aid: enabled “wide-ranging” conversations, opening new and specific (individualized) topics; identified SU interests and resources - Motivating method: interesting to use, modern - Supportive IT portal: supportive, fast, thorough   *Obstacles to use for SU:*   - SU obstacles identified by nurses included: poor mental state, absence of motivation and absence of IT skills   *Obstacles to use for workers:*   - Insufficient resources (time, rooms and IT skills) to get to know and use the intervention - Negative attitude towards the intervention: not part of principal work, perceiving the information available as too brief, not having experience in this role, limited opportunity to practice, valuing face-to-face support once patients discharged over internet-based support |  |
|  |  |  |  |  | (Koivunen, Huhtasalo, Makkonen, VÄLimÄKi, & HÄTÖNen, 2012) Qualitative data from workers  *Nurses’ roles in patient education*   - Education (delivered using IT or leaflets) can support more patient-centred working relationships. - Nurses’ roles occur on a flexible continuum from most patient-centred (learner) to most nurse-centred (limiter): - Being in learner role re IT could reverse conventional roles and cause embarrassment to nurses, or could be viewed as an opportunity to learn more about patient’s needs - Advisors “gently guide the patient” but allow patient to select topics and choose where to go on internet - Limiter role: nurse focused, set limits to patients’ use of the computer and internet   Nurses who used IT psychoeducation more frequently used phrases that indicated a patient-centred role (learner, advisor) than those who used conventional education (47% cw 27% frequency) |  |

Table 1: TIDieR intervention details and key findings

| INTERVENTION/  WHY  TIDieR Q1,2 | MATERIALS  TIDieR Q3 | PROCEDURES & PROVIDER  TIDieR Q4,5 | DELIVERY: HOW, WHERE, WHEN  TIDieR Q6-8 | TAILORING, ADHERENCE  TIDieR Q9-12 | KEY FINDINGS RELATED TO INTERVENTION USE BY SU AND WORKER |
| --- | --- | --- | --- | --- | --- |
| ***Personal Control in Rehabilitation (PCR)***  *Goal:* To facilitate optimal quality and continuity of care and self-management of patients with schizophrenia; to provide informal and formal carers with information about treatment  *Theory:* PCR expected to give patients more control of their illness (support health monitoring) & treatment, increase contact with caregivers, provide info about treatment and improve continuity of care. Expected to lead to reduced face-to-face contacts and increased digital and telephone contacts. Provided informal caregivers with tailored treatment information and gave workers access to evidence-based treatment information. | (de Leeuw, van Splunteren, & Boerema, 2012)   - Open portal and three protected, personalized portals, for SU, informal caregivers and formal caregivers. - Includes SU self-management tools (making appointments, information on treatment options, psycho-education, self-tests, questionnaires and log book), and evidence based care programs for caregivers - Digital contact with peers and caregivers (email and chat) | - Patients and their (in)formal caregivers were trained to use PCR - Portals protected by a user name and password, open for use at any time - Help available from a psychiatric nurse for using PCR during the project - Workers were formal caregivers in mental health organizations | - SU obtained treatment information, used self-management tools, added information to portal, communicated with caregivers/peers, granted portal access to informal caregivers/relatives - Workers accessed evidence-based treatment guidelines, communicated with SU about treatment plans and answered SU questions - Two mental health organizations, Netherlands, 2009-2011 | - Safety problems caused a break of 2 months in the study, with lower use of the website after this break - Average of 41 site visits per day at the end of the study (all users). In one site, number of site visits by SU and formal caregivers were equivalent. Visits by informal caregivers were much lower | (de Leeuw et al., 2012) Qualitative data from SU and workers  *SU experiences:*   - Before trial, SU expected improved communication with caregivers; some concerned about privacy and reduced face-to-face contact - After 9 months, perceived communication advantages: able to ask questions any time; more accurately formulate problems; receive better help - Technical aspects of PCR needed to be improved (e.g. text size) - Perceived PCR focussed too much on illness and treatment and needed more personal content   *Worker experiences:*   - Initially concerned that using computers and internet could raise SU paranoia and reduce face-to-face contact - After 9 months, considered PCR to be time consuming and additional activity as PCR not integrated with other systems; concerned about missing SU messages - Appreciated modern communication method, increased their use of email, increased SU control in treatment decisions; worker able to respond more quickly if “worse functioning” signalled - Considered information in website reliable and good to use |

|  | | | | | |
| --- | --- | --- | --- | --- | --- |
| INTERVENTION/  WHY  TIDieR Q1,2 | MATERIALS  TIDieR Q3 | PROCEDURES & PROVIDER  TIDieR Q4,5 | DELIVERY: HOW, WHERE, WHEN  TIDieR Q6-8 | TAILORING, ADHERENCE  TIDieR Q9-12 | KEY FINDINGS RELATED TO INTERVENTION USE BY SU AND WORKER |
| ***Mental Health Engagement Network (MHEN)***  *Goal:* to electronically link individuals to their mental health care professionals, and to enable clients to take an active role in their health maintenance using embedded tools. Needed to make mental health care system sustainable.  *Theory:* promoting “health and early intervention through a smart technology model of care” (Forchuk et al., 2014, p.2). Promoting SU-worker information sharing, SU access to personal information and tools. | (Forchuk et al., 2013)   - Web-based Electronic health record including care and crisis plans - Embedded tools including: SU Mood Monitor; health journal notes; prompts to assist in daily living; tracking physiological measures such as blood pressure and weight - Secure mental health care professional messaging | - SU given half-day training - Smartphone and health record made available to SU - Additional weekly drop-in training or at-home training in special cases - Blog with training details and resources for SU and workers - Workers were mental health care professionals with at least 8 eligible clients on their caseload | - SU accessed health record including medications, family history, care plans, provider details - SU used phone functions to set-up prompts for appointments and treatment - SU used web tools: mood monitor, health journal, prompts and reminders, physiological measures tracking, messaging provider - Health records updated daily - Participating workers had access to health record and could message SU - Four community mental health agencies, Canada, 2012-2104 | - 16,928 visits to health record page over 12 months - The health journal (record) was the most frequently used component (20,883 hits to page) - At 12 months, 93% of participants surveyed were using the smartphone and 45% were currently using the health record. Smartphone was used to contact care provider by 77%. Accessing the health record was 7^th^ most common use of smartphone. - SU use of the health record decreased over time. | (Forchuk et al., 2014) Qualitative data from SU and workers  *SU experience: Benefits*   - Improved access to workers and impacted information access and sharing - Increased self-awareness and connection to society - Felt in greater control of health management and able to function more independently   *Barriers*   - Sign-in process onerous; application speed; confusion about web-based app - Lack of engagement of some providers - Design features were a barrier for some clients: speed, screen size & sensitivity   *Worker experience: Benefits*   - SU more accessible and communication more consistent - Increased SU self-esteem, connectedness and self-reflection - Pleased that SU could manage appointments and organize personal health goals - Saved time in case management   *Barriers:*   - Checking messages onerous, sign-in process slow, felt that solving clients’ technical problems was not their role |
|  |  |  |  |  | (Forchuk, Donelle, Ethridge, & Warner, 2015)  *Benefits:*   - At 12 months, SUs used their smartphones for various health and non-health related activities. Largest health use was to contact care providers. Participants also used the phone to communicate with family and friends.   *Barriers:*   - SU perceived the smartphone to outperform the online personal health record 12 months after the intervention - Perceptions of helpfulness of the personal health record and having access to a personal crisis plan decreased over time, as did use of the record as mental health improved |

| INTERVENTION/  WHY  TIDieR Q1,2 | MATERIALS  TIDieR Q3 | PROCEDURES & PROVIDER  TIDieR Q4,5 | DELIVERY: HOW, WHERE, WHEN  TIDieR Q6-8 | TAILORING, ADHERENCE  TIDieR Q9-12 | KEY FINDINGS RELATED TO INTERVENTION USE BY SU AND WORKER |
| --- | --- | --- | --- | --- | --- |
| ***CommonGround (CG)***  *Goal:* to increase client involvement in treatment decisions  *Theory:* Founded on principles of person-centred care, choice, collaborative goal setting and informed shared decision-making. Shared decision making contributes to self-determination by placing treatment decisions in context of client’s goals, values and preferences.  (Deegan, 2008) | (Deegan, 2010; Salyers et al., 2017)   - Web-based interactive tools to prepare for medication appointment and support recovery actions. - Includes survey with personal goal (Power statement) and personal medicines, database, recovery library, CG report, shared decision report, personal data exploration, peer videos, recovery strategies - Touchscreen computer kiosk used to complete a one page ‘health report’ prior to appointment with prescriber, which highlights SU selected discussion topics - Peer worker available for technical and emotional support before and after appointment | - CM worked with SU to develop a Power Statement and personal medicines and enter in software - SU completed questions on touchscreen prior to appointment with prescriber, with PW support - CG report created: PW helped to review if needed - Prescriber discussed CG report and treatment decisions with SU. Report updated - CM or PW helped SU implement plan and use CG tools between medication visits. - Workers involved were prescribers, nurses, peer workers, case managers | - SU and workers had unique views of website, however information was fully transparent to SU and authorized users - CG website accessible at clinic, home or library. - Used by SU over 4 months in 2006-2007 (Goscha & Rapp, 2015) and for 18 months in 2013-2015 (Bonfils et al., 2016) - Community mental health centre, Kansas, USA (Goscha & Rapp, 2015) - Four sites of an urban community mental health service, Indiana, USA (Bonfils et al., 2016) | (Goscha & Rapp, 2015)   - No modifications indicated - Study purposefully included participants who were highly engaged and participants who did not appear to be engaged. Criteria for these groups were not outlined. | (Goscha & Rapp, 2015) Qualitative data from SU and workers  Differences between SU who were highly engaged versus not engaged   - *Positive prior working relationship with prescriber:* Highly engaged SU’s had positive relationships with their prescriber who offset initial fears about using the intervention. CG enhanced communication, with SU increasing their intervention use over the year to identify goals, share concerns and to be involved in decision making. SU who did not have a prior positive relationship did not change their involvement in decision making. - *Presence of a meaningful goal supported shared decision making:* Engaged SU had identified a meaningful goal that was acknowledged by the provider. Making progress towards goals supported more active participation in decision making. SU’s with low engagement did not have a meaningful goal reflected in CommonGround report or prescriber notes. - *Discussion about previously undisclosed information*: Highly engaged SU discussed more information and disclosed previously undisclosed information in consultations - *Familiarity with concept of personal medicine*: Highly engaged SU and their providers familiar with personal medicines and used these to explore options beyond medicine - *Case manager helped SU to engage in and follow through shared decision making:* Highly engaged SU had a case manager who was actively involved in all phases of shared decision making |
|  |  |  |  | (Bonfils et al., 2016)   - Implementation coach supported implementation - 65% of participants completed a health report at least once over 20 months. - CommonGround Fidelity Scale used to assess fidelity. A moderate fidelity level assessed at 6 and 12 months. - PW was not employed full time for whole study period, which limited some SU opportunity to use CG | (Bonfils et al., 2016) Qualitative data from workers  *Benefits of the program:*   - Breadth and relevance of material - Peer videos valued as being “normalizing”, enabling clients to “see that they’re not the only one” (p.3) - CG described as a nonthreatening platform that “opened the door to productive conversations” (p.4) - Improved treatment consistency by providing resources to staff that covered gaps in their knowledge and bridging staff changes - Improved client preparation for their appointment and ensured that client’s goals were being addressed - Peer specialists helped SU to be comfortable talking to clinicians   *Barriers to implementation:*   - Complexity of intervention required staff time and added to busy schedule - Need for training new staff was given low priority - Staff or clients not being computer/tech savvy, some clients intimidated - Difficulty using system that does not interface with electronic medical record - Unreliable internet connectivity; difficulty using computers in home visits - Lack of fit with existing service structure, e.g. appointment schedule; not fitting with “crisis-driven setting where focus was on addressing immediate needs” (p.4) - Full program not implemented/poor integration into typical work: e.g. unchanging power statements, inconsistent recording of shared decisions, uneven team and SU participation, staff turnover and intervention not available every day. |

| INTERVENTION/  WHY  TIDieR Q1,2 | MATERIALS  TIDieR Q3 | PROCEDURES & PROVIDER  TIDieR Q4,5 | DELIVERY: HOW, WHERE, WHEN  TIDieR Q6-8 | TAILORING, ADHERENCE  TIDieR Q9-12 | KEY FINDINGS RELATED TO INTERVENTION USE BY SU AND WORKER |
| --- | --- | --- | --- | --- | --- |
| ***Momentum (pilot study)***  *Goal:* A smartphone application to support shared decision making and to reflect importance of supporting recovery  *Theory:* partnership model stated (cited Cochrane systematic review) for shared decision making approach to interaction between worker and SU. SDM challenges paternalism and asymmetry of power between workers and SU. SDM is empowering and part of recovery-oriented practice.  (Korsbek & Tonder, 2016) | (Korsbek & Tonder, 2016)   - Smartphone application, including interactive activities to identify central topics for next appointment and appointment evaluation tool. Tool to develop and evaluate coping strategies - Website for prescriber access, not linked to electronic health records - Peer support for implementation. | - Peer workers introduced intervention through workshops and information for SU and workers - Delivered by multidisciplinary staff and doctors - Intervention use by consumers and providers was supported by 2 peer consultants - Workers were multidisciplinary staff: nurses, occupational therapists, psychologists and social workers (n = 12) and doctors (n = 7) | - SU used app and shared preparations that they wanted worker to see on website. - Administration site matches SU to worker. - SU used app to answer questions to decide which topics to discuss in future meetings, then to evaluate the meeting - Worker could download the app and be a registered user, to view prepared material shared by consumer - Used by SU and workers over a 4 month pilot study - Long-term rehabilitation ward, service for young people with psychosis and a mental health community centre, Denmark, year implemented not stated | - Adherence not stated - Purposively selected SU who used Momentum. No information about the number of non-users | (Korsbek & Tonder, 2016) Qualitative data from SU and workers  *Usability:*   - App was useful, easy to navigate with relevant content - Use was highest when workers found the app useful; some workers (especially doctors) did not review SU information prior to appointments - Workers found app supported people to remember what they wanted to discuss, provided an overview, enabled elaboration of SU chosen topics - SU and workers valued access to coping strategies - Lack of integration with existing IT and log-in procedure were barriers to use - Some workers expressed concern that consumers may be “too poorly functioning” to use the app - Workers needed help from peer workers to use the technology   *Consumer participation and influence:*   - Workers “considered app a means to greater consumer involvement” (p.170). - SU valued being able to set own agenda for meetings - Preparing for meeting and sharing preparation with worker considered important by SU and workers, however some SU “did not dare to share their preparation with the treatment team” (p.171)   *Quality of treatment consultations*   - Meeting preparation provided conversation starters and facilitated better conversations with staff - 4/7 SU valued having ready access to coping strategies and their own coping strategies becoming integrated into treatment |

| INTERVENTION/  WHY  TIDieR Q1,2 | MATERIALS  TIDieR Q3 | PROCEDURES & PROVIDER  TIDieR Q4,5 | DELIVERY: HOW, WHERE, WHEN  TIDieR Q6-8 | TAILORING, ADHERENCE  TIDieR Q9-12 | KEY FINDINGS RELATED TO INTERVENTION USE BY SU AND WORKER |
| --- | --- | --- | --- | --- | --- |
| ***Blended Flexible Assertive Community Treatment (Blended FACT pilot study)***  *Goal:* to support patients with everyday activities (self-management) through an internet portal and to add online communication option for interaction with nurse  *Theory:* Internet-based interventions can empower people experiencing mental illness to manage their own health. People using ACT require frequent support to manage daily tasks. Tele-psychiatry may meet this need. Portal developed using a platform for the development of guided internet interventions: mijnTherapie.  (Blankers, van Emmerik, Richters, & Dekker) | - Telepsychiatry: computer, internet connection, webcamera and skype installed in patients’ homes - Internet platform to support patients with everyday activities using educative videos, information, a leisure activities bulletin board, agenda to schedule appointments with the nurse and web forum to establish contact with peers | - SU informed about portal in recruitment meeting - Computer, internet and webcam installed in SU home. - SU given access to myMentrum website - Workers were psychiatric nurses | - SU contact nurses using Skype at scheduled times - SU contact nurses instantly during office hours using text-chat - Schedule appointments with nurses through myMentrum - SU watch educative videos, review bulletin board with leisure activities, contact peers through forum. No information about worker use of these features - Used by SU and workers over a 3 month pilot study - SMI treatment centre, Netherlands | - Average of 5.4 Skype contacts per month with nurses (range 0-17) - Large variation in SU use of myMentrum platform | (Blankers et al.)  Qualitative data from SU  Patient experiences and adverse events   - Patients liked using Skype for contact with nurses, other patients, friends or relatives - Four patients experienced log-in to platform as complicated - Overall treatment satisfaction was between satisfactory to good: patient satisfaction with the blended intervention was no different to satisfaction with care as usual (as hypothesized) |

| INTERVENTION/  WHY  TIDieR Q1,2 | MATERIALS  TIDieR Q3 | PROCEDURES & PROVIDER  TIDieR Q4,5 | DELIVERY: HOW, WHERE, WHEN  TIDieR Q6-8 | TAILORING, ADHERENCE  TIDieR Q9-12 | INTERVENTION/  WHY  TIDieR Q1,2 |
| --- | --- | --- | --- | --- | --- |
| ***ReConnect***  *Goal:* To support service user involvement in care, servicer-user provider collaboration, and personal recovery (Gammon et al., 2017)  *Theory:* Designed to support recovery-oriented practice in mental health. CHIME recovery processes (Leamy, Bird, Le Boutillier, Williams, & Slade, 2011) referenced;  assessed recovery-orientation using (Le Boutillier et al., 2011) 4 practice domains.  (Gammon, Strand, & Eng, 2014) | (Gammon et al., 2014)   - Web-based interactive tool box including ‘what is important in my life’ for recording values; ‘life domains’ to describe current situation and supports/barriers; ‘my medications’ list; ‘network map’ to show relationships; ‘exercises’ to strengthen life skills; ‘crisis management’ relapse prevention plan; ‘monitoring’ daily life such as sleep, nutrition; ‘goals and activities’ to work towards; ‘good to know’ platform and illness information; ‘my helpers’ contact information; ‘’forum’ and ‘diary’ personal notebook. - Peer forum for anonymous peer connection - Text messaging to/from provider | - Two hours of group, dyad or individual training provided to SU and workers at outset and as requested. - Monthly face-to-face ReConnect Cafes for service users facilitated by a service user consultant. - SU and worker invited to use in whatever way may benefit their working relationships - SU given secure log in - Designed for non-emergency use only - Workers were mixed health professions, mostly women over 40 years old and with median 10 years experience in mental health | - SU determined how to use the website and decided which providers had access. - SU could message worker through portal and use forum to communicate with peers - Providers sent messages and/or responded to messages from SU, could view modules with SU, followed SU progress and provided feedback on SU content - Used where and when chosen by SU or worker, for 6 months - Two communities, Norway | - Dyads determined how to use - Use of portal across dyads was highly diverse - Median number of SU log-ins over study = 17 (range 1-151) - Median number of messages sent by SU over study = 2 (range 0-43) - Toolbox: Tool use by SU was overall low. Modules not used by most participants “could be frequently used and valued by one or two participants” (Gammon et al., 2017, p.6). - Forum: median number of SU visits was 21 (range 0-364); 35% of SU were active forum users (> 10 posts) - 70% of workers answered secure messages from SU - Workers answered a median of 6 messages (range 1-27) - Both groups reported that 6 months was not long enough to “learn and optimally adapt” their use of the internet portal | (Gammon et al., 2017) Qualitative data from SU and workers  Recovery-oriented practice  *Personally defined recovery*:   - SU and worker valued that by using the modules SU’s gained an overview of their lives and greater sense of direction   *Working relationship*:   - SU identified that life domain and goal/activity modules helped collaboration with providers become more focused on their needs - Some SU were frustrated by workers who did not interact with them using ReConnect, instead saying how busy they were - Some SU appreciated increased flexibility in meetings relative to in person meetings that were sometimes described as unnecessary or unproductive - Some providers expressed concerns about pressuring service users to use ReConnect and creating added SU burden   *Organizational commitment*   - Workers reported barriers to committing to using ReConnect including technical infrastructure barriers and workload concerns |
|  |  |  |  |  | (Strand, Gammon, Eng, & Ruland, 2017) Qualitative data from SU and workers *Theme 1: New relational avenues:* ReConnect enriched working relationships in the following ways:   - - *Ownership:* SU took on a more active role, particularly by using modules to write down their goals and plans   - *Common Ground*: secure and asynchronous communication through the portal facilitated SU transfer of everyday thoughts and emotions not usually discussed and timely help from workers   - *Goals and direction*: working on goals became more concrete and substantial, giving dyads a greater sense of direction   - *Sense of presence and availability*: portal facilitated a sense of extra time and support in SU lives.   *Theme 2: Out of alignment*: difficulties experienced in working together through ReConnect included:   - *Initiative and responsibility:* Perceptions about who should initiate and be responsible for using ReConnect in work together were mis-matched. - *Waiting for the other*: a lack of worker response could lead to SU feelings of mistrust and loss of meaning in portal use. A lack of SU response could generate worker frustration. - *Feeling overwhelmed*: workers felt overwhelmed by frequent messages, ambiguities about how to respond therapeutically and added workload - *Clarifications and agreements*: workers identified that making explicit agreements about use of the portal would have made the collaboration easier and reduced uncertainties |

| 1. INTERNET-BASED INTERVENTIONS USED WITH WORKER EMPLOYED IN RESEARCH | | | | | |
| --- | --- | --- | --- | --- | --- |
| INTERVENTION/  WHY  TIDieR Q1,2 | MATERIALS  TIDieR Q3 | PROCEDURES & PROVIDER  TIDieR Q4,5 | DELIVERY: HOW, WHERE, WHEN  TIDieR Q6-8 | TAILORING, ADHERENCE  TIDieR Q9-12 | KEY FINDINGS RELATED TO INTERVENTION USE BY SU AND WORKER |
| ***Health Technology Program (HTP)***  *(Brunette et al., 2016)*  *Goal:* To provide in-person, individualized relapse prevention planning incorporating technology-based treatments with guidance from a case manager in outpatient community mental health settings  *Theory:* Using technology to “provide flexible delivery of evidence-based treatments that include guided support of mental health professionals” (p.1035). Relapse prevention planning based on stress-vulnerability coping model; SDM  (Baumel et al., 2016(Brunette et al., 2016)) | - FOCUS: interactive smartphone illness self-management system: coping strategy training and brief interventions. - 5 modules cover coping with symptoms, mood, sleep, social situations and managing medication. - A daily support and psychoeducation website for participants and family members, including web-based moderated forums - Two web-based CBT self-administered education sessions for psychosis: coping with voices, coping with paranoia - Computerized clinical decision support system used by participant and prescriber to support evidence-based pharmacology - Participants given a Smartphone with FOCUS and if needed, a laptop computer to access other tools | - HTP timeframe was 6 months beginning within 60 days of hospital discharge - MHTC worked in tandem with prescribers - Brief manual and worksheets support plan development and sharing - MHTCs received weekly supervision from site’s program directors and bi-weekly supervision re technology issues and relapse plan implementation - Workers were mental health technology coaches (MHTC): trained case managers with one year experience working in mental health | - Meetings scheduled between participant and MHTC - Phase 1: Plan development - MHTC and participant developed initial relapse prevention plan - Participant decided who else to involve in the plan and selected health technology tools to use with MHTC support - Phase 2: Implementation - MHTC provided monitoring and support: e.g. taught relapse prevention skills, could view client entries on FOCUS app and provided live consultation, tracked progress with digital CBT programs - Participants could message MHTC if needed who would respond during business hours - Phase 3: Plan modified, MHTC supported transition to usual clinical services after 6 months - Ten mental health services, USA, 2013-2014 | - Average number of contacts with MHTC was 28.2 over 6 months, or on average, one 39 minute consultation per week - 36% of contacts took place in the clinic and 35% were technology-mediated (phone calls, text messages, email) - 52% of contacts were case management focused, followed by discussion of relapse prevention plan in 44% of contacts - 87% of participants used at least one digital tool - 85% used FOCUS - 59% used the web-based CBT education sessions - 56% used daily support site - 45% of contacts with MHTC involved discussion of digital tools | (Baumel et al., 2016)  Data from participant self-report survey   - 72% of participants were satisfied with the intervention - Integrating digital tools with human support was found to be acceptable and feasible for this population |

| INTERVENTION/  WHY  TIDieR Q1,2 | MATERIALS  TIDieR Q3 | PROCEDURES & PROVIDER  TIDieR Q4,5 | DELIVERY: HOW, WHERE, WHEN  TIDieR Q6-8 | TAILORING, ADHERENCE  TIDieR Q9-12 | KEY FINDINGS RELATED TO INTERVENTION USE BY SU AND WORKER |
| --- | --- | --- | --- | --- | --- |
| ***Self-Management And Recovery Technology (SMART pilot study)***  *Goal:* To promote personal recovery using digital resources that could be incorporated into mental health service consultations using a tablet computer  *Theory:* Personal recovery based on CHIME recovery processes (Leamy et al., 2011) and self-management based on cognitive behavioural therapies, with opportunity to learn from shared lived experience. The intervention is hypothesized to effect personal recovery by positively influencing self-stigma and self-efficacy  (Thomas, Farhall, Foley, Rossell, et al., 2016) | - Website with lived experience accounts (videos) of personal recovery, additional videos of clinician and family members’ experiences, and academic expert and consumer leader information. - Website content themes: recovery, managing stress, health, me, relationships, empowerment, life - Peer-moderated online forum. - Text and reflective exercises and tools in content areas to complement videos - Worker brought tablet computer to meetings to access SMART | - Participant and worker met for up to eight x 50min face-to-face sessions held over 3 months - Worker set up account and showed participant how to access website to facilitate use outside session - Used alongside treatment as usual - Workers were trained facilitators from the community mental health sector | - Participant and worker collaboratively selected content from the site, shared video viewing and discussed website material - Discussion included: reflecting on personally relevant content, considering changes the participant may wish to enact and setting goals for coming week (p.6) - Worker encouraged participant to use website between sessions, complete reflective exercises or make forum posts - Participants from community mental health and residential services, Australia, 2015 | - All participants completed 8 sessions - Website was used in 76/80 of attended sessions (95%) - 6/10 (60%) logged on to website between sessions with worker, with a median of 4.5 log-ons per participant - 6/10 (60%) posted public comments on the site | (Thomas, Farhall, Foley, Leitan, et al., 2016)  Data from questions about participant experience with the website   - Integrating the website as a tool in sessions “functioned well” (p.7) - Some participants expressed that they would have been less engaged without the sessions with the worker - Some participants commented that using the site facilitated discussion with the worker, for example: Using the website *“kept our discussions going in a direction that we wanted to go in”* and helped to *“cover things in more depth than I would have by myself”(p.8)* |

| INTERVENTION/  WHY  TIDieR Q1,2 | MATERIALS  TIDieR Q3 | PROCEDURES & PROVIDER  TIDieR Q4,5 | DELIVERY: HOW, WHERE, WHEN  TIDieR Q6-8 | TAILORING, ADHERENCE  TIDieR Q9-12 | KEY FINDINGS RELATED TO INTERVENTION USE BY SU AND WORKER |
| --- | --- | --- | --- | --- | --- |
| ***Personalized Real-Time Intervention for Motivation Enhancement (PRIME)***  *Goal:* to improve motivation and functioning in people with recent-onset schizophrenia-spectrum disorders  *Theory:* Motivational and cognitive challenges can occur among people experiencing schizophrenia. Using digital technology that can be accessed frequently may circumvent these challenges and improve motivated behaviour in the early phases of the illness. Refers to self-determination theory.  (Schlosser et al., 2016) | - Mobile app intervention with supportive online environment - Evidence-based micro interventions provided by coaches, based on CBT, mindfulness and psychoeducation - Text-message based motivational coaching - Individualized goal setting - Social networking via direct peer-to-peer messaging - Community “moments” feed to capture and reinforce rewarding experiences and goal achievements - Consumers given Smartphone if they did not own one, with app access | - Two stages: stage 1 was a pilot, stage 2 was start of an RCT. Modifications made to frequency of contact from coaches after stage 1 - Participant was provided with Apple iPhone, PRIME installed - Meeting with coach for set-up session to orient participant to app and discuss goals participant would like to achieve by using PRIME. - User profile created on first sign-in. Goals and interests categorized in domains of health/wellness, social, productivity, creativity - Participant chose if profile was public (shared with PRIM community), or private - Daily challenges set that supported goal-related activities; achievements could be recorded in the app - Participants encouraged to use app daily with minimum frequency of 1/week over 12 weeks - Coaches had 2 hour training using manual and weekly case review meetings - Workers were “motivation coaches”: Masters-level clinicians experienced in CBT | - Participant created a public or private profile - Participant selected and documented progress on small, self-determined goals in health/wellness, social relationships, creativity and productivity. Posted goal achievement or spontaneous positive moments to site - Participant sent messages to other users and viewed public profiles of other members - Workers explained their role and engaged consumer in text-based chat to determine their goals - Workers “reached-out” to participants through the app at least 4 days per week - Workers used evidence-based microinterventions drawn from CBT, mindfulness and psychoeducation to help overcome obstacles that hinder goal progress - Workers could review profiles and member posts - Participants were from early psychosis clinic and community treatments services, USA, year of implementation not disclosed | - Once app set-up, participant selected personally tailored daily challenges associated with goals - Frequency of contact from worker was tailored according to participant preferences and progress - All participants were retained in the trial, with an average log in rate of 3 – 11 times per week - The most frequent user-initiated action was liking a post from a peer - Consumers undertook on average 19 challenges over the 12 weeks (1.5 per week), with most occurring in the health/wellness domain - Challenge completion rate was 80% - On average, participants interacted twice per week with coaches and twice per week with peers | *(Schlosser et al., 2016)*  Data from participants   - Mean overall consumer satisfaction with PRIME was 8/10 - Most popular PRIME feature was the ability to comment on users’ posts and least popular was the ability to view the coach profiles - Participants interacted with coaches through comments, likes and messages, with messages used most - Log ins and engagement in interaction with coaches increased in stage 2 when coaches sent more frequent, shorter personalized messages in more casual tones |

| INTERVENTION/  WHY  TIDieR Q1,2 | MATERIALS  TIDieR Q3 | PROCEDURES & PROVIDER  TIDieR Q4,5 | DELIVERY: HOW, WHERE, WHEN  TIDieR Q6-8 | TAILORING, ADHERENCE  TIDieR Q9-12 | INTERVENTION/  WHY  TIDieR Q1,2 |
| --- | --- | --- | --- | --- | --- |
| ***PeerTECH (Pilot study)***  *Goal:* To teach older adults with SMI and chronic medical conditions how to self-manage medical and psychiatric conditions  *Theory:* Older people experiencing SMI have challenges to remain living independently in the community. Integrated medical and psychiatric self-management programs, delivered by peer specialists using technology to standardize and guide users, has the potential to expand reach of SM interventions.  (Fortuna et al., 2017) | - Smartphone app that includes: access to personalized self-management support, intervention components matched to consumer’s needs and goals, medication reminders and a chat feature - In-person eModules with peer-led videos and text on psychoeducation and coping skills training, viewed with CPS on a tablet computer. - Each participant provided with Smartphone and 3 month data plan | - Peer specialists completed 16 hour PeerTECH training and met with peer supervisor once per week. - PeerTECH eModules reviewed with Peer Specialists in 10 x weekly one hour sessions. Sessions occurred in participants’ home over 3 months. - Smartphone app reinforced skills learned in in-person session - Peer specialist sent text messages to participants on average 3 times/week - Workers were certified peer specialists | - Participant reviewed educational eModules with CPS in their home - CPS sent text messages to consumers during the week - Participants were living in the community and recruited from case managers’ caseloads in a clinical team, USA, year of implementation not stated | - Two (20%) participants dropped out of the study - Two participants experienced initial difficulty using the App - 80% participants completed 10 or more in-person sessions - 74-88% of participants engaged weekly with the App; 33-47% engaged daily - On average participants completed 42% of all self-management tasks over intervention course - Peer specialists shared their personal experiences of managing their own health | (Fortuna et al., 2017)  Data from participants completing questionnaires   - All participants demonstrated an increase in psychiatric self-management skills (change from baseline to post treatment statistically significant (p = <0.001) - No other outcome statistically significant, although some participants demonstrated an increase in medical self-management and other outcomes including self-efficacy - Results in measures of peer support included: 6/8 participants reported increased levels of hope; 5/8 reported increased level of social support; 6/8 reported increased level of empowerment |

Table 2: Intervention: recovery and self-management outcomes – extracted findings

| Intervention | Service Users’ perspectives: Self-management and recovery outcomes | Workers’ perspectives: Self-management and recovery outcomes |
| --- | --- | --- |
| ***Mieli.net*** |  | **Qualitative:** “Nurses experienced the portal as supporting patients’ self-management abilities because it enabled autonomous access to information and could be used independently outside the education sessions” (Anttila et al., 2008, p.150) |
| ***PCR*** | **Qualitative:** “Overall, PCR was evaluated positively by patients as a modern way to communicate, the overview of their treatment goals and medication was reported to facilitate them to communicate and decide about their treatment goals.” (de Leeuw et al., 2012, p.358) | **Qualitative:** “It is also appreciated that patients have more control about treatment decisions, because of easy access to their treatment goals and medications.” (de Leeuw et al., 2012, p.359) |
| ***MHEN*** | **Quantitative:**   1. Perception of Smart Technology Form: Internet-based health record gave participants ‘quite a bit more’ (26.8%) or an ‘extreme amount more’ (21%) independence.   **Qualitative:** “Since adopting this technology, many clients expressed feelings of greater control in their health management and in their life, and that they were able to function more independently.” (Forchuk et al., 2014, p.4) |  |
| ***CommonGround, Kansas*** | **Qualitative:** “Clients who had an important goal guiding discussions in medication clinic also reported a higher degree of control in decision making.” (Goscha & Rapp, 2015, p.268) |  |
| ***CommonGround,***  ***Indiana*** | **Quantitative: (**Salyers et al., 2017)   1. Patient Activation Measure: no significant change from baseline to 18 months 2. Autonomy Preference Index: no significant change from baseline to 18 months 3. Recovery assessment scale (perceived level of recovery): significant change for total scale and “no domination by symptoms” subscale from baseline to 18 months 4. State Hope Scale: no change over time | **Quantitative:** (Salyers et al., 2017)   1. Providers perception of consumer involvement: increased significantly from baseline to 18 months |
| ***Momentum*** | **Qualitative:** “Four of the seven consumer interviewed said that they were happy to have at hand a “catalogue” of things they could do for themselves in difficulty situations.” (Korsbek & Tonder, 2016, pp., p.170)  “More consumers argued that as the preparation was an opportunity for them to set a personal agenda for their next treatment appointment they could thereby influence treatment consultations.” (Korsbek & Tonder, 2016, pp., p.171) | **Qualitative:** “Several staff members considered the app a means to greater consumer involvement…. Another stated: ‘It is important that clients assume ownership of their own treatment. This app can help’” (Korsbek & Tonder, 2016, pp., p.170). |
| ***Blended FACT*** | **Quantitative:**   1. Mental health confidence scale (empowerment): scores improved significantly over time, but there was no difference between the control and intervention groups. |  |
| ***ReConnect*** | **Qualitative:** “Service users reported being helped in gaining an overview of their lives and becoming more conscientious of where they were headed and what kind of help they needed.” (Gammon et al., 2017, p.8) | **Qualitative:** “Health providers in particular described how the portal enabled service users to set the agenda for collaboration by describing, in their own words, their situations, priorities and goals. This in turn strengthened service users’ ownership, or sense of engagement and responsibility, of their everyday recovery processes.” (Strand et al., 2017, p.4) |
| ***HTP*** | No relevant data | |
| ***SMART*** | **Quantitative:**   1. Questionnaire for the Process of Recovery and Recovery Assessment Scale (personal recovery): statistically significant small to medium effect size 2. Schizophrenia Hope Scale: statistically significant medium effect size 3. Friendship Scale (social connectedness): No significant change 4. Internalized Stigma of Mental Illness Scale: small to medium effect 5. Generalized Self-efficacy Scale: no statistically significant effect |  |
| ***PRIME*** | No relevant data | |
| ***PeerTech*** | 1. Herth Hope Index: 6/8 reported increased levels of hope; no statistically significant change 2. Empowerment Scale: 6/8 reported increased empowerment; no statistically significant change 3. Medical Outcomes Study Social Support Survey instrument: 5/8 reported increased levels of social support; no statistically significant change 4. Illness management and recovery scale (psychiatric self-management skills): statistically significant change from baseline to 3 months 5. Self-efficacy for managing chronic disease scale: 6/8 reported improvement, however no statistically significant change |  |

References:

Anttila, M., Koivunen, M., & Välimäki, M. (2008). Information technology-based standardized patient education in psychiatric inpatient care. *Journal of Advanced Nursing, 64*(2), 147-156. doi:10.1111/j.1365-2648.2008.04770.x

Baumel, A., Correll, C. U., Hauser, M., Brunette, M., Rotondi, A., Ben-Zeev, D., . . . Kane, J. M. (2016). Health Technology Intervention After Hospitalization for Schizophrenia: Service Utilization and User Satisfaction. *Psychiatric services, 67*(9), 1035-1038. doi:doi:10.1176/appi.ps.201500317

Blankers, M., van Emmerik, A., Richters, B., & Dekker, J. (2016). Blended internet care for patients with severe mental illnesses: An open label prospective controlled cohort pilot study. *Internet Interventions, 5*, 51-55. doi:10.1016/j.invent.2016.07.004

Bonfils, K. A., Dreison, K. C., Luther, L., Fukui, S., Dempsey, A. E., Rapp, C. A., & Salyers, M. P. (2016). Implementing CommonGround in a Community Mental Health Center: Lessons in a Computerized Decision Support System. *Psychiatric Rehabilitation Journal*. doi:10.1037/prj0000225

Brunette, M. F., Rotondi, A. J., Ben-Zeev, D., Gottlieb, J. D., Mueser, K. T., Robinson, D. G., . . . Kane, J. M. (2016). Coordinated Technology-Delivered Treatment to Prevent Rehospitalization in Schizophrenia: A Novel Model of Care. *Psychiatric services, 67*(4), 444-447. doi:doi:10.1176/appi.ps.201500257

de Leeuw, J., van Splunteren, P., & Boerema, I. (2012). Personal control in rehabilitation: An internet platform for patients with schizophrenia and their caregivers. *Open Journal of Psychiatry, 2*, 355-361. doi:http://dx.doi.org/10.4236/ojpsych.2012.224050

Deegan, P. E. (2010). A web application to support recovery and shared decision making in psychiatric medication clinics. *Psychiatric Rehabilitation Journal, 34*, 23-28. doi:10.2975/34.1.2010.23.28

Forchuk, C., Donelle, L., Ethridge, P., & Warner, L. (2015). Client perceptions of the mental health engagement network: A secondary analysis of an intervention using smartphones and desktop devices for individuals experiencing mood or psychotic disorders in Canada. *Journal of Medical Internet Research Mental Health, 2*(1), e1. doi:10.2196/mental.3926

Forchuk, C., Rudnic, A., Hoch, J., Godin, M., Donelle, L., Rasmussen, D., . . . McKillop, M. (2013). Mental health engagement network (MHEN). *International journal on Advances in Life Sciences, 5*(1-2), 1-10.

Forchuk, C., Rudnick, A., Hoch, J., Donelle, L., Campbell, R., Osaka, W., . . . McKillop, M. (2014). Mental health engagement network: Innovating community-based mental healthcare. *Journal of General Practice, 2*(1).

Fortuna, K. L., Dimilia, P. R., Lohman, M. C., Bruce, M. L., Zubritsky, C. D., Halaby, M. R., . . . Bartels, S. J. (2017). Feasibility, Acceptability, and Preliminary Effectiveness of a Peer-Delivered and Technology Supported Self-Management Intervention for Older Adults with Serious Mental Illness. *The Psychiatric quarterly*. doi:10.1007/s11126-017-9534-7

Gammon, D., Strand, M., & Eng, L. S. (2014). Service users’ perspectives in the design of an online tool for assisted self-help in mental health: a case study of implications. *International Journal of Mental Health Systems, 8*(1), 2. doi:10.1186/1752-4458-8-2

Gammon, D., Strand, M., Eng, L. S., Børøsund, E., Varsi, C., & Ruland, C. (2017). Shifting Practices Toward Recovery-Oriented Care Through an E-Recovery Portal in Community Mental Health Care: A Mixed-Methods Exploratory Study. *Journal of Medical Internet Research, 19*(5), e145. doi:10.2196/jmir.7524

Goscha, R., & Rapp, C. (2015). Exploring the Experiences of Client Involvement in Medication Decisions Using a Shared Decision Making Model: Results of a Qualitative Study. *Community Mental Health Journal, 51*(3), 267-274. doi:10.1007/s10597-014-9759-y

Hoffmann, T.C., Glasziou, P.P., Boutron, I., Milne, R., Perera, R., Moher, D., et al. (2014). Better reporting of interventions: template for intervention description and replication (TIDieR) checklist and guide. *British Medical J.* ;348:g1687.

Koivunen, M., Huhtasalo, J., Makkonen, P., VÄLimÄKi, M., & HÄTÖNen, H. (2012). Nurses' roles in systematic patient education sessions in psychiatric nursing. *Journal of Psychiatric and Mental Health Nursing, 19*(6), 546-554. doi:10.1111/j.1365-2850.2011.01833.x

Korsbek, L., & Tonder, E. (2016). Momentum: A Smartphone Application to Support Shared Decision Making for People Using Mental Health Services. *Psychiatric Rehabilitation Journal, 39*, 167-172.

Le Boutillier, C., Leamy, M., Bird, V. J., Davidson, L., Williams, J., & Slade, M. (2011). What does recovery mean in practice? A qualitative analysis of international recovery-oriented practice guidance. *Psychiatric services, 62*, 1470-1476. doi:http://dx.doi.org/10.1176/appi.ps.001312011

Leamy, M., Bird, V., Le Boutillier, C., Williams, J., & Slade, M. (2011). Conceptual framework for personal recovery in mental health: systematic review and narrative synthesis. *The British Journal of Psychiatry, 199*, 445-452. doi:10.1192/bjp.bp.110.083733

Salyers, M. P., Fukui, S., Bonfils, K. A., Firmin, R. L., Luther, L., Goscha, R., . . . Holter, M. C. (2017). Consumer Outcomes After Implementing CommonGround as an Approach to Shared Decision Making. *Psychiatric services, 68*(3), 299-302. doi:10.1176/appi.ps.201500468

Schlosser, D., Campellone, T., Kim, D., Truong, B., Vergani, S., Ward, C., & Vinogradov, S. (2016). Feasibility of PRIME: A Cognitive Neuroscience-Informed Mobile App Intervention to Enhance Motivated Behavior and Improve Quality of Life in Recent Onset Schizophrenia. *JMIR research protocols, 5*(2), e77. doi:10.2196/resprot.5450

Strand, M., Gammon, D., Eng, L. S., & Ruland, C. (2017). Exploring Working Relationships in Mental Health Care via an E-Recovery Portal: Qualitative Study on the Experiences of Service Users and Health Providers. *JMIR Mental Health, 4*(4), e54. doi:10.2196/mental.8491

Thomas, N., Farhall, J., Foley, F., Leitan, N. D., Villagonzalo, K.-A., Ladd, E., . . . Kyrios, M. (2016). Promoting Personal Recovery in People with Persisting Psychotic Disorders: Development and Pilot Study of a Novel Digital Intervention.(Report)(Author abstract). *Frontiers in Psychiatry, 7*. doi:10.3389/fpsyt.2016.00196

Thomas, N., Farhall, J., Foley, F., Rossell, S. L., Castle, D., Ladd, E., . . . Kyrios, M. (2016). Randomised controlled trial of a digitally assisted low intensity intervention to promote personal recovery in persisting psychosis: SMART-Therapy study protocol.(Self-Management and Recovery Technology)(Report). *BMC Psychiatry, 16*(1). doi:10.1186/s12888-016-1024-1

Välimäki, M., Anttila, M., Hätönen, H., Koivunen, M., Jakobsson, T., Pitkänen, A., . . . Kuosmanen, L. (2008). Design and development process of patient-centered computer-based support system for patients with schizophrenia spectrum psychosis. *Informatics for Health and Social Care, 33*(2), 113-123. doi:doi:10.1080/17538150802127207
